# Assessment of Critical Health and Safety Risks in Homes where Hoarding is Prevalent

**DOI:** 10.1007/s10900-023-01238-0

**Published:** 2023-06-07

**Authors:** Persephone Larkin, Christiana Bratiotis, Sheila R. Woody

**Affiliations:** 1 Department of Psychology, University of British Columbia, 2136 West Mall, Vancouver, BC V6T 1Z4 Canada; 2 School of Social Work, University of British Columbia, Vancouver, BC Canada

**Keywords:** Multidisciplinary, Community, Environmental risks, Delphi method, Hoarding

## Abstract

Hoarding behaviour sometimes requires intervention from community agencies to reduce risks to residents and the nearby community. Human services professionals from a wide range of disciplines are called upon to address hoarding concerns, often in collaboration with each other. No guidelines currently exist to guide staff from those community agencies in a shared understanding of common health and safety risks that occur with severe hoarding behaviour. Using a modified Delphi method, we aimed to generate consensus among a panel of 34 service-provider experts from a range of disciplines on essential risks in the home that would require intervention for health or safety reasons. This process identified 31 environmental risk factors that experts agreed are critical to assess in cases of hoarding. Panelist comments outlined the debates that commonly occur in the field, the complexity of hoarding, and the difficulty with conceptualizing risks in the home. The multi-disciplinary consensus achieved on these risks will facilitate better collaboration between agencies by providing a minimum standard of what to evaluate in hoarded homes to ensure health and safety standards are being met. This can improve communication between agencies, specify the core hazards that should be incorporated into training for professionals who work with hoarding, and facilitate more standardized assessment of health and safety risks in hoarded homes.

## Introduction

Hoarding involves an accumulation of domestic clutter that can severely impair functioning in the home [1]. Examples include high piles of objects at risk of toppling over on someone, narrow pathways that increase the potential of tripping or falling and hinder access for emergency responders, and combustibles such as newspapers or clothing piled near heat sources that can easily ignite a fire [2]. Risks for neighbours with shared walls and other issues make hoarding a social problem that necessitates multidisciplinary community involvement to reduce these health and safety risks [3, 4]. Unfortunately, no standards or guidelines inform providers across disciplines about how to assess basic health and safety risks. This project harnessed the expertise of professionals from an array of specialties to identify the most essential risks that frontline providers should assess in hoarding situations.

Hoarding comes to the attention of human services professionals through a variety of means. Providers discover cluttered situations when visiting the home for reasons unrelated to hoarding or through referrals from housing providers [5]. If conditions in the home violate fire or building codes, then community providers must get involved to rectify the situation and achieve compliance with the codes. However, there are frequently multiple risks to consider, necessitating the cooperation of professionals from various disciplines with unique jurisdictions. In fact, almost 80% of community cases involve more than one agency [6]. Hoarding thus requires a cross-sectoral and multiagency approach to ensure that the home is safe, but agencies often differ in their hoarding assessment approaches and goals. Some regions have created multidisciplinary task forces to reduce risks and achieve health and safety standards. These teams include professionals such as a fire inspector, building inspector, housing provider, mental health professional or older adult services [7]. Team members in these formal and informal collaborations take on responsibilities relevant to their training and job title, while consulting with the rest of the team to target a range of risks in hoarded homes. Importantly, these team members represent different agencies who have various mandates and accordingly prioritize addressing distinct aspects of situations where there is hoarding.

When responding to reports of hoarding, professionals from different disciplines or agencies tend to assess specific aspects of the home that reflect their own training and organizational mandate. For example, Luu et al. [8] reported that the Vancouver-based Hoarding Action Response Team (HART) focused on fire risks in the home because fire prevention officers represented half the team, whereas Metro Housing Boston assisted tenants at risk of losing their housing subsidy and therefore prioritized conditions that would violate lease agreements. Mental health clinicians tend to focus on psychological aspects of hoarding and often neglect formal assessment of health and safety conditions of the home [9]. On a multidisciplinary team, these diverging foci of assessment can generate provider disagreement about the main targets of concern and complicate the task of developing a coordinated intervention plan [7]. Establishing multidisciplinary consensus about key areas of environmental risk in hoarded homes would support multidisciplinary teams in case planning, including prioritizing problems relevant to occupational health and safety for in-home service providers, working with housing providers to preserve tenancy while addressing public health risks, and ensuring safety of vulnerable residents such as frail older adults.

Some teams use established measures of conditions in the home, the most common of which is the Clutter Image Rating (CIR) scale, a pictorial scale of clutter volume in the bedroom, living room, and kitchen [10]. Although the CIR is a practical tool that can be used by anyone, including non-professionals, it only examines clutter volume and does not appear to be sensitive to changes that occur during mental health treatment for hoarding [11]. More importantly, in assessing only the volume of clutter, the CIR does not guide judgments about risks in the home or functional impairment due to clutter. In principle, a home could have clinical levels of clutter volume while still meeting basic health and safety standards. The HOMES Multidisciplinary Hoarding Risk Assessment [7] and the Uniform Inspection Checklist [UIC; 12] are frequently-used checklists that do capture health and safety risks in the home and are good tools to organize a conversation on a multidisciplinary team. Both of these checklists were created within a housing context and thus may not reflect interdisciplinary agreement on the most essential risks in hoarded homes.

To establish such consensus, we convened a multidisciplinary panel of service providers who were highly experienced in working with hoarding in community settings. The aim of the study was to develop a list of the highest-priority health and safety issues that every professional – from any discipline – should assess in homes with hoarding. Such a list would facilitate interprofessional communication and inform intervention planning, particularly interventions oriented toward harm reduction. Preliminary studies have shown that a harm reduction approach can decrease fire risks [13] and support housing retention [13]. A consensually-developed list of health and safety risks also supports less experienced assessors, for whom assessment of hoarded homes can feel overwhelming. This project used the Delphi method to combine the perspectives of experts from different disciplines into a list of the most important environmental risks to assess in hoarded homes. A Delphi study functions like an asynchronous online focus group with multiple rounds. Each panelist provides structured input (in this case, ratings of the importance of assessing different aspects of hoarded homes) and commentary explaining their ratings or suggesting new items. After the first round, subsequent panelist ratings are influenced by anonymized feedback showing how other panelists rated and commented on each item in the previous round. In comparison with a focus group, the Delphi’s asynchronous method and anonymous feedback about other panelists’ opinions encourages more thoughtful reflection and egalitarian participation and may reduce the influence of social desirability on responses.

## Method

### Participants

Delphi surveys typically rely on panelists with established expertise [14], which for this study was defined as service providers based in Canada or the USA who had direct professional involvement in the assessment or intervention of a minimum of five hoarding cases within the past two years. Some exceptions to this inclusion criterion were made for highly-experienced experts who had recently retired after decades of experience with hoarding. As Table 1 shows, the 34 panelists who completed Round 1 worked in a range of sectors, principally allied behavioural health (including occupational therapy and social work), code enforcement, and housing. Attempts were made to recruit panelists from the legal profession and child protection, but these efforts were not successful. These two sectors are less typically involved in cases of hoarding, so it is unusual to find professionals in these disciplines who are highly experienced with hoarding.

**Table 1:** Frequency of Panelists Across Professional Sectors

	Number of Panelists			Round 3%
	Round 1 (*N* = 34)	Round 2 (*N* = 31)	Round 3 (*N* = 30)	
Allied behavioural health	19	17	16	53%
Code enforcement	6	5	5	17%
Housing	5	5	5	17%
Animal welfare	2	2	2	7%
First responder	1	1	1	3%
Professional organizer	1	1	1	3%

On average, the panelists had 11 years of experience with hoarding (*M* = 11.07, *SD* = 7.35), with a range of 2–30 years. The modal panelist (*n* = 14) reported having experience with at least 100 hoarding cases. The least-experienced panelists (*n* = 9) had participated in five to 25 cases. Most panelists (*n* = 26; 76%) were female, with seven male (21%) and one non-binary/third gender (3%) panelist. Panelist age ranged from 29 to 70 years (*M* = 46.29, *SD* = 12.05). Three panelists failed to return for Round 2 (one indicated it was due to time constraints), and one panelist dropped out for Round 3 for the same reason, as shown in Table 1. As there were only four dropouts, neither the demographic profile nor the sector representation of the final panel was greatly affected.

### Procedure

As briefly mentioned earlier, the Delphi method facilitates agreement among experts using repeated iterations of a survey along with controlled feedback in the form of aggregated statistics about panelist ratings and panelist comments [15]. A modified Delphi, which is the approach we took, extracts the content of the initial survey from an extensive literature review [15]. We generated items for the first round of the Delphi from (a) a scoping review that we conducted on community harm reduction targets, (b) structured interviews that the principal investigators conducted with experts in community-based interventions, (c) established measures of risk in hoarded homes, and (d) the investigators’ own expertise based on years of collaborative research on community-based interventions for hoarding.

Initial panelist recruitment efforts were conducted through community organizations and individual service providers known to the researchers; further recruitment was by snowball sampling. Prospective panelists were screened by telephone to confirm expertise and to determine their service sector. Once 40 qualified experts with good cross-sectoral representation had been recruited, the study was launched. Ultimately, 34 panelists started and completed the first round. Before each round, panelists attended a 15-minute videoconference orientation meeting led by one of the investigators and a research assistant. These sessions began with a pre-recorded instructional slide presentation that described the purpose and procedures of the Delphi method and instructions for that round of the survey, using screenshots and animations relevant to each specific round. After the orientation presentation, panelists’ questions were answered, and the link to that round of the Delphi poll was provided. The survey was presented on the Qualtrics platform.

Informed Consent was obtained at the start of the first round. The first round also included questions about panelists’ age, gender, province or state, number of hoarding cases they had seen, and years of experience in their service sector. Each round of the Delphi presented a series of potential health or safety risks relevant to hoarded homes (e.g., blocked stairs, combustibles near heat source), and panelists rated the importance for generalists to assess each item. The emphasis on generalist assessment recognizes that specialists (e.g., fire prevention officers, social workers) will likely use more detailed assessments, and this study aimed to identify risks that every service provider, regardless of discipline, should assess in hoarded homes. Panelists rated the importance of assessing each item using a 1–3 scale: 1 = “critical for every provider to assess”, 2 = “important but not critical”, and 3 = “not a priority for basic health and safety standards”. A textbox for comments was provided for each item so that panelists could provide a rationale for their rating, suggest alternate terminology that would be more typically used in their field, or recommend additional items for assessment.

As is common practice with Delphi studies, three rounds of ratings were solicited [16]. Immediately after each round closed, researchers analyzed the responses and prepared the subsequent round. Items for which panelists showed at least 75% agreement (as critical, important, or not a priority) were eliminated from subsequent rounds, following procedures used in previous literature [17]. In Rounds 2 and 3, each item was accompanied by statistical feedback in the form of a pie chart of panelist response frequencies [18] and panelists’ anonymous comments about the rationale for their ratings in the preceding round [19].

Cognizant of the common problem of panelist attrition across the multiple rounds of a Delphi study [18], we took several steps to retain and engage panelists. Based on the work of Belton et al. [18] and Turnbull et al. [20], we regularly emailed panelists during the recruitment period and between rounds. Each round of the survey was open for two weeks, and panelists who had not yet completed the survey received a reminder after one week. The aim was for participants to feel like part of a team working towards a shared goal. We carefully monitored sector representation as the rounds progressed. The four panelists who failed to complete all three rounds were from sectors that had good representation, suggesting that diversity of perspective was preserved.

## Results

### Round Overview

Round 1 began with 42 health and safety items from the sources described above. Round 2 had 43 items, including 33 items from Round 1 that had not gained consensus as well as three compound items (e.g., “heaters/radiators”) without consensus that were split into multiple simple items (e.g., “heaters” and “radiators”) for Round 2. The final round had 21 items that had failed to obtain consensus in earlier rounds. Each round took, on average, 34–47 min to complete (trimmed means).

In Round 1, panelists showed consensus (defined here as 75% agreement) on six items they judged as “critical” to include in assessment of hoarded homes. Researchers modified the wording of one of these six consensus items in response to panelist comments, so the item was presented again in the second round. In Round 2, panelists achieved consensus on 22 of the 44 presented items, with 10 judged as “critical” to assess, 10 as “important” to assess, and two items judged as not a priority for generalist assessment. Although panelist disagreement on three of the items was stable (changed less than 15%) from Round 1 to Round 2, these risks were presented again in the final round because panelists continued to offer new substantive commentary about the items. In Round 3, 12 of the 21 presented items obtained consensus: seven that were judged as “critical”, four rated as “important” to assess, and one that panelists agreed was not a priority. Panelist disagreement was stable for two items (ratings changed less than 15% from Round 2 to Round 3).

### Priority Assessment Items

Table 2 shows items that panelists agreed were “critical” or “important” to assess in hoarded homes. Generally speaking, the items spanned five themes: fire safety, safe pathways, health and wellness, structural integrity, and poor sanitation. (Items that panelists agreed are “not a priority” to assess are not shown in the table.) As the No Consensus section (bottom of Table 2) shows, panelists unanimously judged four items as a priority (i.e., no panelist thought those four items were “not a priority”), but they disagreed on whether the items are “critical” or merely “important” to assess, suggesting the boundary between “critical” and “important” is a fuzzy one. As represented in Table 2, Fire Safety items tended to have strong and early consensus as critical to assess. Examples of these items include the clearance of combustibles from heat sources.

**Table 2:** Priority Items to Assess Harm Potential in Hoarded Homes

Item	Critical	Important	Not a Priority
**Consensus in Round 1**			
Ability for emergency responders to enter (with equipment)	**97%**	3%	0%
Exterior doors accessible (can open at least 90°)	**85%**	15%	0%
No open flame used as a heat source (e.g., kerosene lantern, barbeque grill, fireplace)	**85%**	15%	0%
Stove has 1-foot (30 cm) clearance and oven is clear	**85%**	15%	0%
Smoke detectors work and have 1.5 feet (45 cm) clearance	**82%**	15%	3%
Heaters/radiators have 1-foot (30 cm) clearance	**79%**	18%	3%
**Consensus in Round 2**			
No combustible items (e.g., clothing, cardboard) beside or on top of heat source	**94%**	6%	0%
Hazardous materials (e.g., fireworks) properly stored	**87%**	13%	0%
Main pathways within daily living spaces are at least 3 feet (90 cm) wide	**87%**	13%	0%
Fireplace has 1-foot (30 cm) clearance or is disabled	**87%**	10%	3%
Space heater (if present) has 3-foot (91 cm) clearance	**84%**	16%	0%
Medications accessible	**84%**	16%	0%
Medical equipment can be used	**81%**	19%	0%
Heating is usable in winter	**81%**	19%	0%
Floor boards, stairs, porch are stable	**81%**	13%	6%
No evidence of electrical problems (e.g., overloaded circuit)	**77%**	23%	0%
No evidence of insect or rodent infestation	6%	**90%**	3%
No spoiled or rotting food	6%	**90%**	3%
Absence of mold, mildew, or chronic dampness	10%	**87%**	3%
No contaminated objects (e.g., used toilet paper or tampons)	13%	**81%**	6%
Roof does not leak	13%	**81%**	6%
Extension cords are not coiled or under combustible materials (e.g., clothing, cardboard)	13%	**81%**	6%
Interior doors passable (can open at least 90°)	6%	**84%**	10%
No evidence of plumbing problems (e.g., clogged drain, leak)	0%	**90%**	10%
No excessive garbage build-up	0%	**90%**	10%
No water damage on floors or walls (e.g., caving walls)	3%	**77%**	19%
**Consensus in Round 3**			
Furnace has clearance	**97%**	3%	0%
Radiators have clearance	**93%**	7%	0%
Stairs are safely usable	**93%**	3%	3%
Carbon monoxide (CO) alarms work and have 1.5 feet (45cm) clearance	**87%**	10%	3%
Access to usable toilet	**83%**	17%	0%
Hallways allow emergency egress	**83%**	17%	0%
In-home care services can be provided	**80%**	20%	0%
Electricity is functioning	23%	**77%**	0%
No standing water	0%	**80%**	20%
No visible water leaks	13%	**80%**	7%
Stacks or piles no more than 4 feet (120 cm) high	20%	**77%**	3%
**No Consensus (Round 3)**			
Electrical appliances and cords are in good condition (no exposed or frayed wires)	**60%**	**40%**	0%
Sleeping room(s) have unobstructed emergency exits	**57%**	**43%**	0%
Hot water tanks have clearance	**37%**	**63%**	0%
No visible urine or feces	**37%**	**63%**	0%
Sink is usable	3%	**73%**	**23%**
Hot water is functioning	3%	**63%**	**33%**
Bathtub/shower is usable	0%	**57%**	**43%**
Kitchen appliances (refrigerator, freezer, stove, oven) are usable	0%	**47%**	**53%**
Access to safe and secure sleeping space	7%	**20%**	**73%**

Note: Items listed here represent the final wording developed through panelist comments and researcher deliberation. Some items do not add to 100% due to rounding

Other items shifted across rounds as panelists read feedback about how other panelists had rated the items and their rationale for these ratings. Between rounds, the researchers used panelist comments to edit items for the subsequent round, which resulted in strong agreement about most items, but panelists continued to disagree about five items. “Access to safe and secure sleeping space” is an example of an item in this category. This item was intended to reflect assessment that the client has a place to sleep, and in Round 1, the item was stated as, “Can sleep in a bed”. Panelists debated this item, however, with some arguing that, “Some clients choose not to sleep in their beds”. After considering the comments, researchers edited the item to the current wording of “access to safe and secure sleeping space”, which most panelists judged as not a priority for assessment, reflecting their commitment to clients’ right to exercise autonomy in how they live.

### Panelist Comments

Items that obtained consensus in the first round were all relevant to well-established fire safety risks. Comments in subsequent rounds often pointed to potential consequences of ignoring certain risks in the home. For example, panelists remarked on less well-known fire risks, such as those related to clearance from “obvious heat/ignition source[s]” or electrical cords (e.g., “Compressed cords can start fires because there is no space for the heat to dissipate. This has killed people in hoarded homes.”). Panelists also commented on items relevant to health, especially the importance of a functioning toilet: “Persons in hoarded homes that cannot access the toilet will go in the home no doubt, which poses health risks for all”. Related to clear pathways, panelists commented on what is required for emergency response personnel to enter the home, such as, “minimum space needed to get stretcher in; firefighters with gear” and “height of stacks are not an issue unless they are in the direct path to egress/usable to rooms”. A distal health-related concern focused on access to in-home care services, such as “I spend countless hours trying to get supports who then refuse to go in [the home] due to conditions … These lack of supports very often lead to death or hospitalization.” These responses described panelists’ experiences of some conditions in hoarded homes that, if left unaddressed, present a possibility of severe consequences.

Panelists’ disagreements about some items showed the complexity of assessing hoarding. One example was about unobstructed sleeping room windows, which can provide a “quick escape in the event of a fire wherever household members sleep”. Although some panelists noted the importance of this item for emergency exit, other panelists did not think having clear access to a window was essential, “if windows could not be used as exits” such as in high-rise apartments. Some items reflecting risk of tripping or falling also provoked disagreement, with some panelists pointing to the importance of clear pathways (e.g., “critical because of the risk of falls”) and others viewing pathways as, “not critical, as paths can be widened, items pushed out of the way, as needed”.

Obviously, even if assessors have a list of items to assess, they will still need to use their professional judgment and consider the context of the situation in the home, and panelist comments reflected some of these considerations. For example, whether stacks or piles of items would present a serious risk depends on the “sturdiness” and “stability” of the stacks or the weight of items in the piles. Similarly, some pests, such as bedbugs, were seen as representing a higher public health risk than others, such as fruit flies. Urine or feces in the home generated commentary around the volume and whether it involved animal or human waste. Overall, panelist comments suggested they viewed higher amounts of animal waste as important, but *any* amount of human waste was considered critical.

Panelists raised concerns about qualifiers such as “clear”, “safe”, or specific distances like, “3 feet of clearance” present in the items. Some panelists did not like clearance measurements (e.g., “tripping up on the 3 feet again - ‘has sufficient clearance’ might be better”), whereas others advocated for this specificity (e.g., “3 feet clearance to any ignition source – heater, fireplace, stove, baseboard heater, etc.”). One panelist remarked that stairs only need to be clear enough for certain tasks such as, “clear enough for emergency access and movement, but not completely clear”. In response to comments like these, items presented in Table 2 were edited to indicate safe movement through the home (e.g., “stairs are safely usable” rather than “stairs are clear”). Notably, “safe” is still a relatively vague description that some panelists did not agree with, illustrating the need for training and experience to properly assess health and safety conditions in the context of each client and their particular vulnerabilities.

## Discussion

This project used the Delphi method to identify the most important health and safety risks for service providers from any discipline to assess in hoarded homes. A multidisciplinary panel of experts achieved early and strong consensus that assessment of fire safety concerns is critical in the context of hoarding, due to the potential for fatalities as well as regulatory (fire code) requirements. Fire safety concerns include not only conditions that raise the risk of fire ignition but also egress for occupants in case of a fire (e.g., exterior door swing) and access for emergency personnel to enter the home for rescue or fire suppression. Safe pathways inside the home, including through living areas and on stairs were also rated as critical to assess because of the potential for tripping or falling. This early and strong agreement may indicate that fire safety and mobility within the home are more common knowledge among service providers who work with hoarding. In contrast, other items reflecting topics such as structural integrity or poor sanitation (sometimes referred to as squalor) may be less widely discussed in the field. As such, they required more conversation and consultation among panelists before agreement was achieved. Furthermore, health and wellness items may be more dependent on the resident’s context (e.g., age, physical condition), in comparison to fire safety risks, which are more universally relevant.

Consensus was our priority for this project – to identify what professionals from a range of disciplines should assess in relation to health and safety in hoarded homes. Overall, panelists showed at least 75% agreement on the rating for 37 of 46 total items, an agreement rate that is comparable to earlier studies [21, 22]. Even among the items that did not achieve 75% agreement, panelists agreed that four of the 10 items are a priority for assessment; they disagreed on whether the items are “critical” or “important” to assess. The level of consensus was likely facilitated by the high quality of panelists’ comments; many noted that they changed their mind because of the rationales articulated by other panelists. For instance, a frequent rationale that panelists offered in Rounds 2 and 3 was something like, “swayed by comments”.

Some health-related items (e.g., ability to access and use medications and medical equipment) had strong panelist consensus as critical to assess. Other items, however, such as access to a usable sink or ability to use kitchen appliances, provoked discussion about the appropriateness of assessing such items in a general community context. These tend to be more subjective lifestyle risks situated in the goals of the client, thereby meaning if the client does not share these goals, then assessing them is not essential. This is particularly relevant in hoarding cases, as the resident is usually not seeking an assessment or assistance. Only about 5% of cases are initiated through self-referral [5], so most of these assessments are conducted due to external pressure such as an eviction risk due to lease violations, fire inspections, or inability to provide necessary in-home support services. Some panelists – perhaps those from an allied behavioural health profession – rated these quality of life items as important to assess. Other panelists – perhaps those who work in tenancy preservation programs – argued that these items are not a priority in the context of residents who have not necessarily invited the assessment.

Panelists also touched on a larger debate about what community service providers have the legitimate authority to assess or require in a client’s home. Which risks *must* be addressed relates to enforcement power, local legal standards, whether the resident owns the property and what type of housing it is (e.g., presence of neighbours with shared walls). In the absence of laws or regulations, such as fire or building codes or terms of a lease agreement, service providers may lack authority to require that health or safety problems be addressed (so there is no point in assessing those items). Some professionals are charged with protecting vulnerable residents and carry a mandate to assess and enforce health and wellness standards for such persons (or animals), but there is more ambiguity in these judgments than in something like building or fire codes.

Addressing problems related to hoarding in community settings can be ethically complex. Although the home is normally a private space, hoarding can lead to serious risks. Competent adults do have a right to make risky behavioural choices, but that right is not unlimited. When hoarding increases the risks for neighbours or the risk of damage to a rented property, discussion turns to what circumstances would permit intervention even against the person’s wishes. The Delphi panelists debated this point in their comments to each other, noting that underlying their judgments of what risks need to be assessed in a hoarded home is the distinction between a basic standard of living required to meet legal codes versus what constitutes a sufficiently severe risk to infringe upon the client’s autonomy in their own home. As one Delphi panelist stated, “I think safety and health take critical precedence over comfort and quality of life in early [harm reduction]”. Although social services and mental health professionals are often interested in the client’s quality of life, being unable to sleep in a bed or prepare food in the kitchen does not require or even justify immediate or involuntary intervention [23]. Many would argue that inability to sleep in a bed does not represent a risk severe enough to compromise the client’s autonomy. On the other hand, imminent fire poses a danger not only to the resident but also to neighbours and first responders and would therefore supersede the right to autonomy in the home. The tensions resulting from this ethical consideration were obvious in the comments and ratings of the panelists when determining what items were “critical” to assess.

In Delphi studies, the quality of the panelists is more important than the sheer size of the panel [24]. Our panel was comprised of highly-experienced professionals from several service sectors that are most commonly involved in hoarding cases. First responders and code enforcement have been well represented in previous research on hoarding in community settings, as fire code violations, clearance from heat sources, and access to fire exits are well-known concerns. Housing providers and inspectors are also frequently represented as they encounter poor sanitation or clutter outside of the home. Previous research on community-based interventions for hoarding has not typically featured allied behavioural health professionals, who tend to focus more on the individual’s functioning in the home, including access to medication, ability to receive home-based health services, or tripping hazards. Including panelists from all these settings gave us a well-rounded view of the problem and facilitated agreement amongst multidisciplinary groups of professionals who can often disagree when working together on complex cases.

Although the panel represented the relevant professional sectors well, panelists were confined to a few jurisdictions in Canada and the USA, which may limit the generalizability to other countries with different rules and regulations. Although the panel’s strong representation of allied behavioural health professionals ensured the relevance to mental health providers, it may also have shifted favour toward items important to these providers. Furthermore, some of the panelists were known to each other and may have discussed their responses outside of the Delphi process (despite being asked not to do so) or may have swung their answers toward the majority because of the presence of well-known experts on the panel (even though comments were anonymous). Similar to other studies [17] we did not obtain complete consensus, which raises the possibility that some panelists may have been reluctant to step out of their specialty stance. For instance, someone from code enforcement may have had more difficulty appreciating the importance of assessing a personal health concern such as ability to access medications.

This project represents a list of the highest priority assessment items and enriches the knowledge base about important issues to assess in hoarded homes. Professionals from some disciplines, such as occupational therapy or fire prevention, tend to have clear protocols for assessing the client or conditions in the home, but these protocols are usually specific to a single human service sector. In hoarding cases, multidisciplinary expertise and cross-disciplinary coordination is necessary to know what to look for in order to target interventions appropriately and to address the most critical health and safety concerns. This Delphi study is the first to establish expert consensus on the highest-priority environmental risk items to assess in hoarded homes. We are currently engaged in the next step in this research, that of collaboratively developing a psychometrically strong measure of the severity of health and safety risks in hoarded homes. Rarely do providers learn about necessary intervention targets and mandates from disciplines outside their own. Outlining the potential risks and educating providers about how to judge these areas is essential to ensure that no critically important areas are missed, regardless of disciplinary training or professional expertise, when assessing conditions in the home.
