# Correction to: Assessment of Critical Health and Safety Risks in Homes where Hoarding is Prevalent

**DOI:** 10.1007/s10900-023-01285-7

**Published:** 2023-10-04

**Authors:** Persephone Larkin, Christiana Bratiotis, Sheila R. Woody

**Affiliations:** 1 Department of Psychology, University of British Columbia, 2136 West Mall, V6T 1Z4 Vancouver, BC Canada; 2 School of Social Work, University of British Columbia, Vancouver, BC Canada

**Correction to: Journal of Community Health**

10.1007/s10900-023-01238-0

The article “Assessment of Critical Health and Safety Risks in Homes where Hoarding is Prevalent” written by Persephone Larkin, Christiana Bratiotis, Sheila R. Woody was originally published electronically on the publisher’s internet portal on 7-June 2023 without open access. With the author(s)’ decision to opt for Open Choice the copyright of the article changed on 1 August 2023 to © The Authors 2023 and the article is forthwith distributed under a Creative Commons Attribution 4.0 International License, which permits use, sharing, adaptation, distribution and reproduction in any medium or format, as long as you give appropriate credit to the original author(s) and the source, provide a link to the Creative Commons licence, and indicate if changes were made. The images or other third party material in this article are included in the article’s Creative Commons licence, unless indicated otherwise in a credit line to the material. If material is not included in the article’s Creative Commons licence and your intended use is not permitted by statutory regulation or exceeds the permitted use, you will need to obtain permission directly from the copyright holder. To view a copy of this licence, visit http://creativecommons.org/licenses/by/4.0.

